# Death of Professor Sewall

**Published:** 1845-07

**Authors:** 


### DEATH OF PROFESSOR SEWALL.

This eminent physician, and pure philanthropist died at Washington City, on the 10th of May last, in the fifty-ninth year of his age. Dr. S. was connected with many of the benevolent enterprises of the day, and had a reputation, wide as his country, for zeal in sustaining every cause by which human happiness may be promoted. His death is a loss, not only to his profession, but to the public at large. We copy from the Medical Examiner the following brief notice of his life:

"Dr. Sewall was a native of Augusta, in the State of Maine. He graduated at the Medical School of Boston, and about the year 1820 removed to the City of Washington, where his talents and acquirements, with an upright deportment and great urbanity of manners, soon procured for him the respect and patronage of a large portion of the inhabitants. He was appointed Professor of Anatomy in the Medical School of that place on its first organization, and continued to discharge the duties of the Chair, with distinguished ability, until the time of his death." Y.

